# Tunable plasmonic substrates with ultrahigh *Q*-factor resonances

**DOI:** 10.1038/s41598-017-16288-3

**Published:** 2017-11-22

**Authors:** Hamid T. Chorsi, Youngkyu Lee, Andrea Alù, John X. J. Zhang

**Affiliations:** 1 Thayer School of Engineering, Dartmouth College, Hanover, NH 03755 USA; 2 Electrical and Computer Engineering, University of Texas at Austin, Texas, 78712 USA

## Abstract

Precisely tailored plasmonic substrates can provide a platform for a variety of enhanced plasmonic applications in sensing and imaging. Despite the significant advances made in plasmonics, most plasmonic devices suffer critically from intrinsic absorption losses at optical frequencies, fatally restricting their efficiency. Here, we describe and engineer plasmonic substrates based on metal-insulator-metal (MIM) plasmon resonances with ultra-sharp optical transmission responses. Due to their sharp transmission spectrum, the proposed substrates can be utilized for high quality (*Q*)-factor multi-functional plasmonic applications. Analytical and numerical methods are exploited to investigate the optical properties of the substrates. The optical response of the substrate can be tuned by adjusting the periodicity of the nanograting patterned on the substrate. Fabricated substrates present *Q*-factors as high as ∼40 and refractive index sensing of the surrounding medium as high as 1245 nm/RIU. Our results indicate that by engineering the substrate geometry, the dielectric thickness and incident angle, the radiation losses can be greatly diminished, thus enabling the design of plasmonic substrates with large *Q* factor and strong sensitivity to the environment.

## Introduction

Fueled by the exciting opportunities offered by strong light-matter interactions, the field of plasmonics experienced an explosive growth in the last two decades, which empowered unsurpassed performance in deep-subwavelength concentration of light^1–3^. Significant progress has been made not only in device technologies such as plasmonic lenses^4,5^, plasmonic nanosensors and biosensors^6,7^, metamaterials and metasurfaces^8–10^, photovoltaics^11^, and near-field imaging^12–14^, but also in fundamentals of optical physics such as nonlinear optical resonances^15,16^, exotic constructive and destructive interferences^17–19^, optical beam manipulation^20–22^, and even optical cloaking and invisibility^23^, to name but a few. However, while offering extreme light confinement, plasmonic structures are challenging in terms of loss and greatly suffer from intrinsic absorption losses due to interband and intraband transitions in the visible and infrared regions, respectively. In general, the optical losses of plasmonic nanostructures are due to damping collision losses, which comprise of Landau damping (absorption loss) and radiative damping (radiation loss). A number of strategies for loss mitigation (or prevention) are being considered. These involve using heavily-doped semiconductor materials such as silicon^24,25^ and GaAs^26^, lowering the temperature which drastically reduces the collision frequency^27,28^, exploiting high-refractive index dielectric resonators^29,30^, using innovative fabrication techniques to reduce the surface roughness^31,32^, and using gain-medium to amplify the resonant emission of surface plasmons^33,34^.

Plasmonic substrates are arrays of plasmonic nanostructures and nanoholes that enable plasmon localization and intensity enhancement along the surface^35^. Engineering the optical response of surface plasmons through designing inventive plasmonic substrates is a crucial step in realizing the next generation of optoelectronic and biomedical devices^36–39^. In particular, multifunctional plasmonic substrates can provide multiple optical functionalities e.g. optical filtering, sensing, and surface-enhanced spectroscopy on a single chip, significantly miniaturizing optoelectronic and biomedical devices^40,41^. Such multifunctional devices require multi-resonance transmission spectra along with high quality factor (*Q*-factor) resonances in order to be efficiently exploited. *Q*-factor determines the width and the strength of a resonance and can be diminished by plasmon loss channels such as surface roughness. Besides the aforementioned loss mitigation mechanisms for plasmonic structures, tuning geometrical parameters is another degree of freedom to lessen the damping collision losses by properly engineering the system geometry. Although, *Q*-factor does not explicitly depend on the geometry (for localized surface plasmon resonances (LSPRs), $Q={\omega }_{SP}/2\gamma$, where ${\omega }_{SP}$ is the LSPR frequency,$\gamma \approx \text{Im}{\varepsilon }_{m}(\omega )/{\partial }_{\omega }\mathrm{Re}{\varepsilon }_{m}(\omega )$ is the plasmon decay rate, ${\varepsilon }_{m}$ is the metal permittivity, and ${\partial }_{\omega }$ indicates derivative with respect to frequency^42^), it implicitly enters to the equation by modifying the ${\omega }_{SP}$.

To date, several multifunctional and multi-resonance plasmonic substrates have been realized^41,43–46^. Although ultra-high *Q*-factors have been reported theoretically or using loss mitigation techniques for plasmonic nanostructures, such *Q*-factors have not been experimentally obtained at room temperature for plasmonic substrates, due to the influence of realistic losses, disorder, and inhomogeneities. In this paper, we propose a geometrically engineered multi-resonance, multifunctional plasmonic substrate with ultra-narrow linewidth resonances operating at the room-temperature. Due to its ultra-sharp resonances, the substrate can be used in a variety of enhanced plasmonic applications. We show both numerically and experimentally that the bandgap resonances of the substrate can be tuned by modifying the periodicity of the nanograting. It has been also shown that the spatial and spectral properties of these resonances can be tailored by changing the nanograting periodicity. In order to demonstrate the versatility of the substrate, refractive-index sensing of the surrounding medium has been carried out. The influences of different parameters including the thickness of the metallic and dielectric grating and the incident angle are investigated in details. The proposed device can operate at both visible and near-IR spectral regions.

## Principle

**Figure 1:**
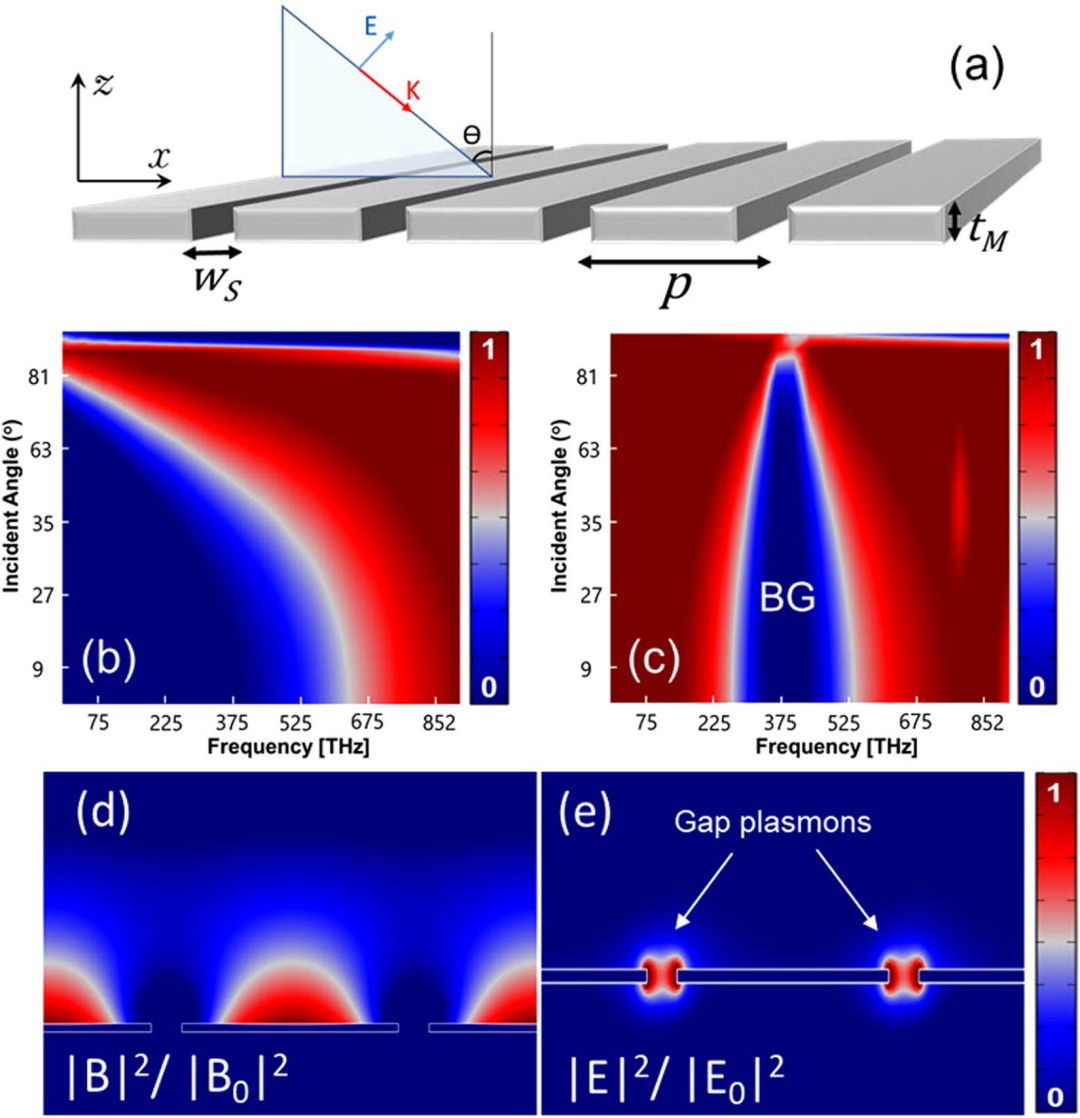
Optical response of a single-layer silver film with nanogratings in air. (**a**) The structure of a thin film with periodic nanogratings, the structure is excited via a TM polarized wave with *θ* incident angle, *p* is the period. (**b**) Transmission optical response of a thin silver film without nanogratings with respect to the incident angle. (**c**) Angular transmission of a silver film with plasmonic nanogratings. The bandgap response can be observed in the spectrum. (**d**,**e**) Magnetic and electric field intensity of a thin film with nanogratings. The excited gap-mode plasmons at the bandgap frequency are depicted.

The principal idea in this paper is to exploit the coupled resonance modes of a thin metal film with nanogratings to obtain sharp bandgap (i.e. near-zero transmission over a narrow spectral range independent of the incident angle) responses. For impinging transverse-magnetic (TM) light this can be obtained by using horizontal waveguide modes. To demonstrate this, a numerical study has been performed to investigate how nanogratings in thin film would affect the light transmission. Numerical simulations were performed via COMSOL Multiphysics (a finite element method (FEM) based software) in the frequency domain. Figure 1(a) shows plasmonic nanogratings in a thin silver film in which *p* is the period, *W*_*s*_ is the slit width, and *t*_*M*_ is the film thickness.

Figure 1(b) presents the transmission optical response of a single-layer silver film with respect to the incident angle at different frequencies. The optical properties of silver is characterized with the Lorentz–Drude dispersion model^47^. As predicted by the classical optics, for optical frequencies the metal film is semi-transparent, i.e., for frequencies higher than the plasma frequency the wave can tunnel through the film, for lower frequencies, however, the silver becomes more “metallic”, which corresponds to a high reflectivity. In contrast, the thin film with periodic nanogratings, represents a bandgap response which is independent of the incident angle. Figure 1(c) depicts the numerical simulations of the angular transmittance of the silver nanograting with *t*_*M*_ = 8 nm and *p* = 220 nm, respectively. It can be observed that for *t*_*M*_ $\ll$λ most of the optical energy is transmitted through the nanograting but because of the coupled resonance modes inside the horizontal channels, as shown in Fig. 1(d ,e ) the metallic grating film presents a strong bandgap response at the resonance frequencies.

**Figure 2:**
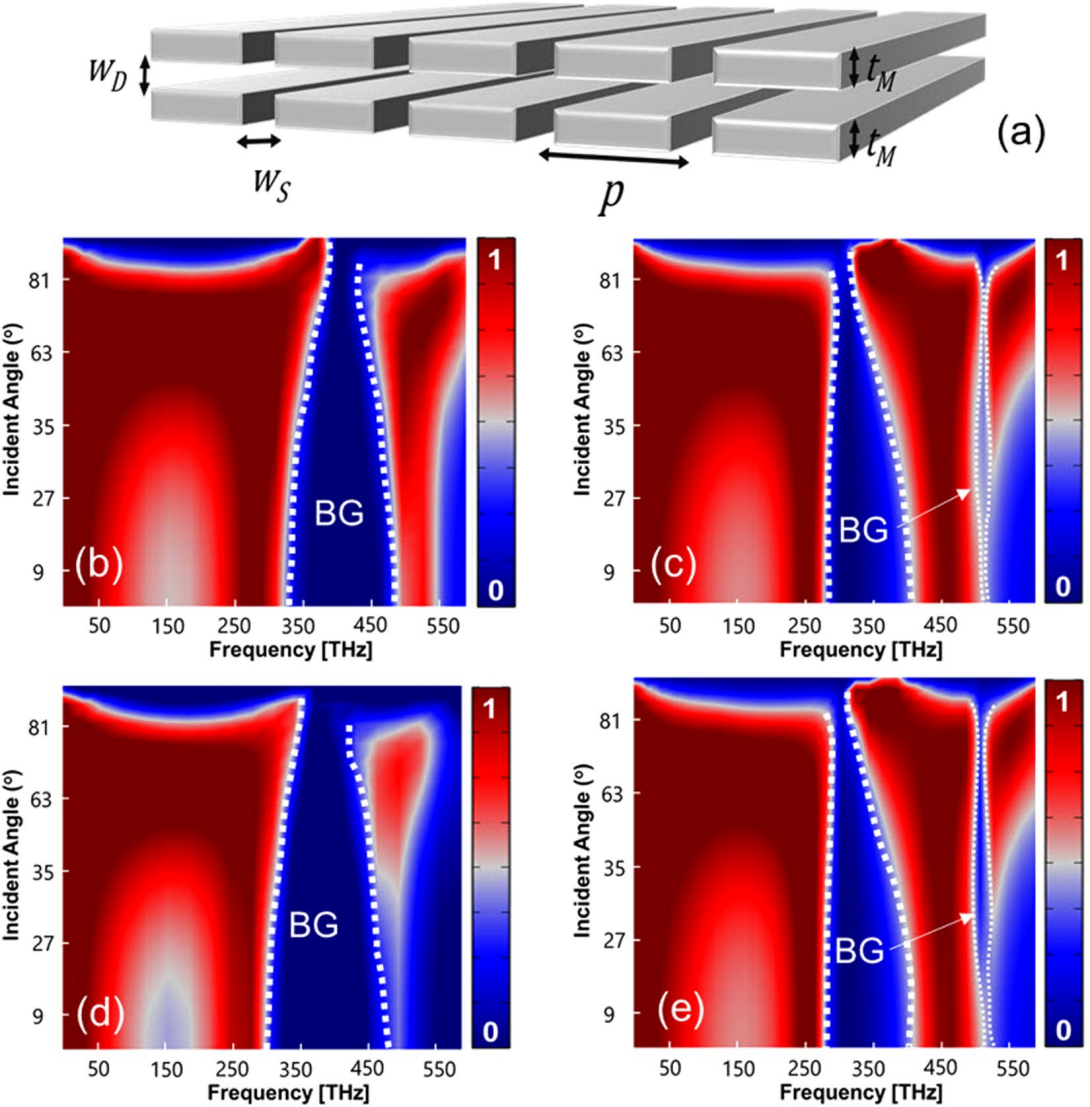
Double-layer plasmonic nanograting in air. (**a**) Dielectric gap with *W*_*D*_ thickness positioned between two silver thin films. (**b**,**c**) Numerical simulation results of the optical transmission spectra for *w*_*s*_ = 40 nm, *t*_M_ = 100 nm, *p* = 180 nm, *w*_D_ = 10 nm, and *w*_D_ = 5 nm respectively. (**d**,**e**) Corresponding analytical results obtained via transmission line theory.

Figure 2, in general, presents a unique phenomenon in which, by placing two plasmonic thin film nanogratings face to face with proper distance W_D_ (dielectric thickness), double resonance transmission bandgap responses can be shaped.

Results also show that the bandgap responses are independent of the incident angle, although their linewidths largely depend on the incident angle. A good agreement between the numerical (top row) and analytical transmission line modeling^14,48^ (bottom row) results can be noticed. Electric and magnetic field intensity results for the double-layer structure in Fig. 2 are illustrated in *Supplementary S1*.

**Figure 3:**
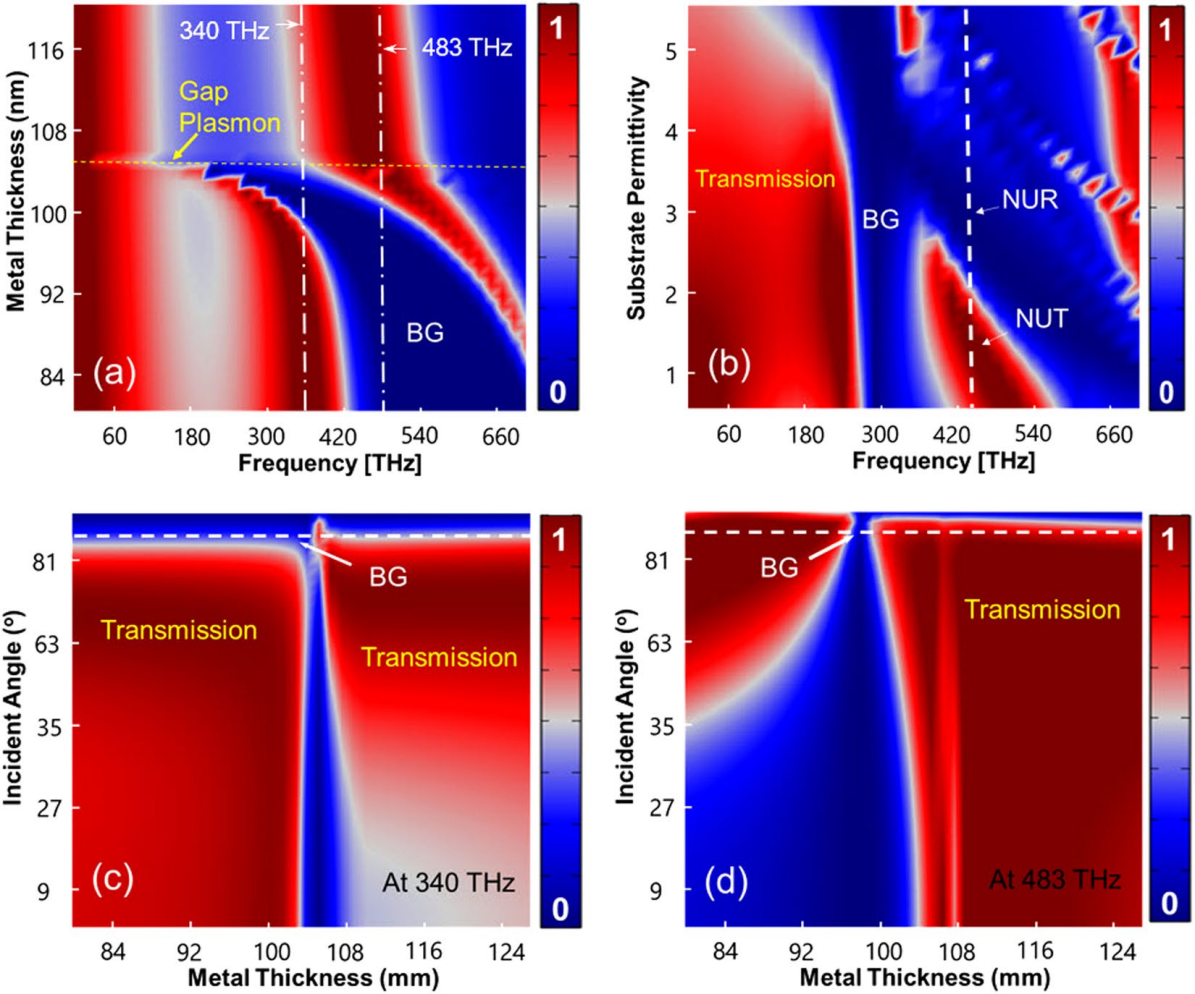
Plasmonic substrate parametric study with p = 180 nm. (**a**) Transmission spectrum of the plasmonic substrate with regard to the metal thickness and GSPs formation. The substrate is excited via $\theta =86$°. (**b**) Optical transmission response of the 220 nm-plasmonic nanograting substrate with regard to the relative permittivity of the substrate. (**c**,**d**) Angular transmission of the substrate against metal thickness at 340 THz and 483 THz, as for part (**a**), respectively. High Q-resonances can be obtained by engineering the metal thickness and the incident angle.

To further investigate the formation of ultra-sharp bandgap responses in double-layer plasmonic substrates, the transmission spectrum of a double-layer silver film with respect to metal thickness is plotted in Fig. 3(a) for p = 180 nm substrate when the substrate is excited normally from the top at $\theta =86$°. Gap surface plasmons (GSPs) are formed at the horizontal dashed line indicated in Fig. 3(a) upon augmentation of the insulator layer. For metal thickness values above the GSP line, the two metal layers are in direct contact with each other, thus no bandgap response can be perceived. Decreasing the height of the metal with respect to the boundary (equivalent to increasing the insulator thickness) by varying t_M_ and retaining the total thickness *W*_*D*_ + 2_*tM*_ constant, initiates the bandgap response. The resonance is blue shifted by further increase of the insulator thickness. These results exhibit that the plasmonic bandgap establishment primarily depends on the insulator thickness but insignificantly on the permittivity of the insulator layer (see *Supplementary S2*).

Unlike the insulator permittivity which does not significantly affect the optical transmission response, the substrate permittivity does affect the transmission spectrum, providing another degree of freedom to engineer the bandgap response. Figure 3(b) represents the transmission spectrum of the plasmonic substrate with p = 220 nm. It can be seen that the choice of substrate can switch the optical response from near-unity transmission (NUT) to near-unity reflection (NUR) at the same frequency. To get an insight into the bandgap formation, the angular transmission plots of the plasmonic nanograting substrate with p = 180 nm are presented at 340 THz and 483 THz in Fig. 3(c) and (d), respectively. Note that even though the bandgap responses persist independent of the incident angle, their linewidths can be engineered by optimizing the incident angle and the metal thickness.

## Results and Discussion

**Figure 4:**
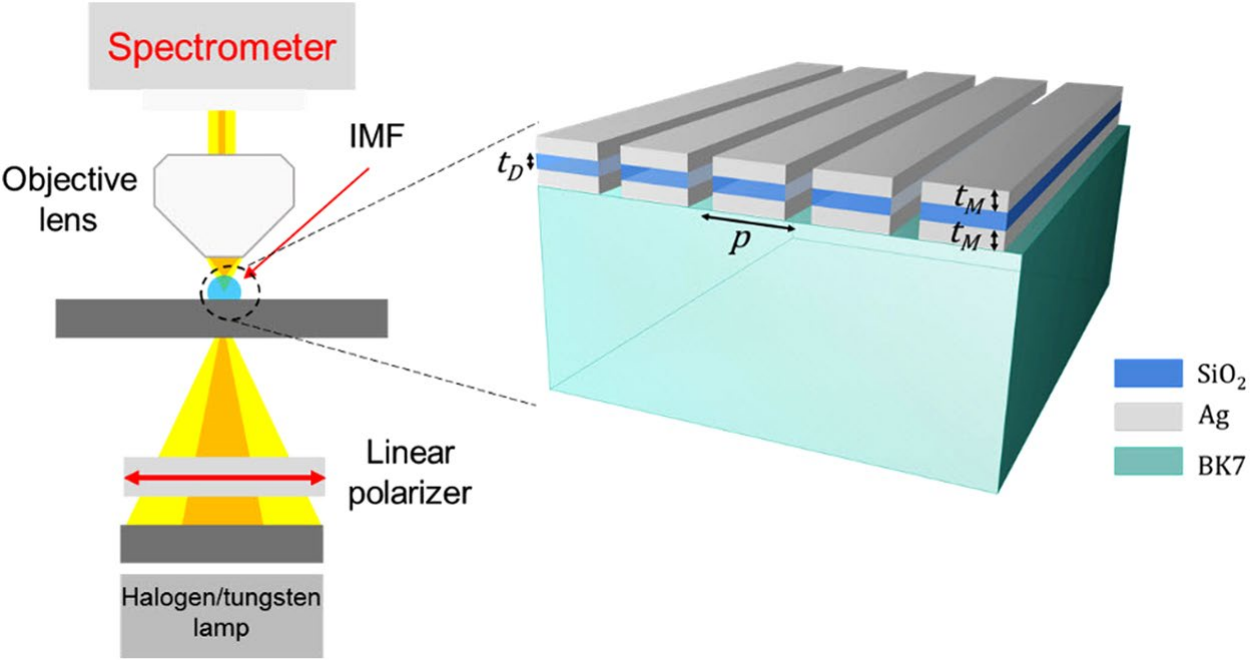
Schematic of the fabricated plasmonic substrate along with the experimental setup for the measurements. Both the substrate and objective lens are immersed in index-matching medium (IMF, n = 1.52). the optimum geometrical parameters obtained from the full-wave numerical results based on the same procedure explained in the previous section are t_M_ = 50 nm, t_D_ = 80 nm and p = 220 nm.

Based upon the engineering procedure described in the previous section, here we realize an experimental demonstration of the bandgap mechanism in the Visible range. Figure 4 displays a schematic of the fabricated substrate and the corresponding optical setup (see methods). The plasmonic substrate is comprised of Ag/SiO_2_/Ag stacks mounted on a BK7 glass with t_M_ = 50 nm, t_D_ = 80 nm, and p = 220 nm obtained via full-wave numerical results to obtain ultra-sharp bandgap responses in the Visible wavelength.

**Figure 5:**
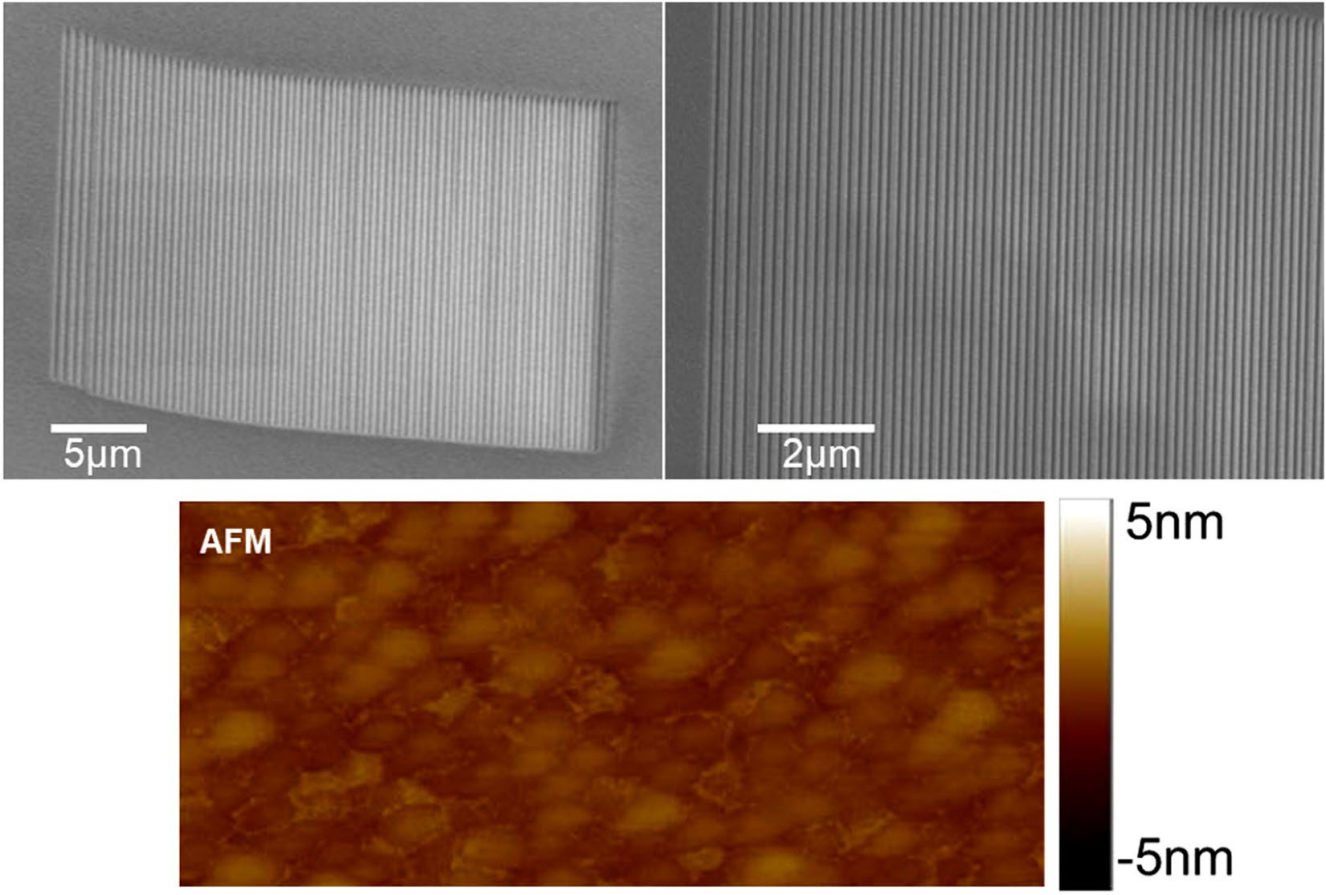
SEM images of the fabricated plasmonic substrate. t_M_ = 50 nm, t_D_ = 80 nm, and p = 220 nm. AFM surface topography of Ag/SiO2/Ag film evaporated on BK7 glass with average roughness of 1.6 nm.

Representative scanning electron microscope (SEM) images of the fabricated substrate with p = 220 nm are presented in Fig. 5. The realized plasmonic nanograting was defined in an MIM film using focused ion beam (FEI SEM/FIB dual beam, FEI Corp., OR, USA) milling.

**Figure 6:**
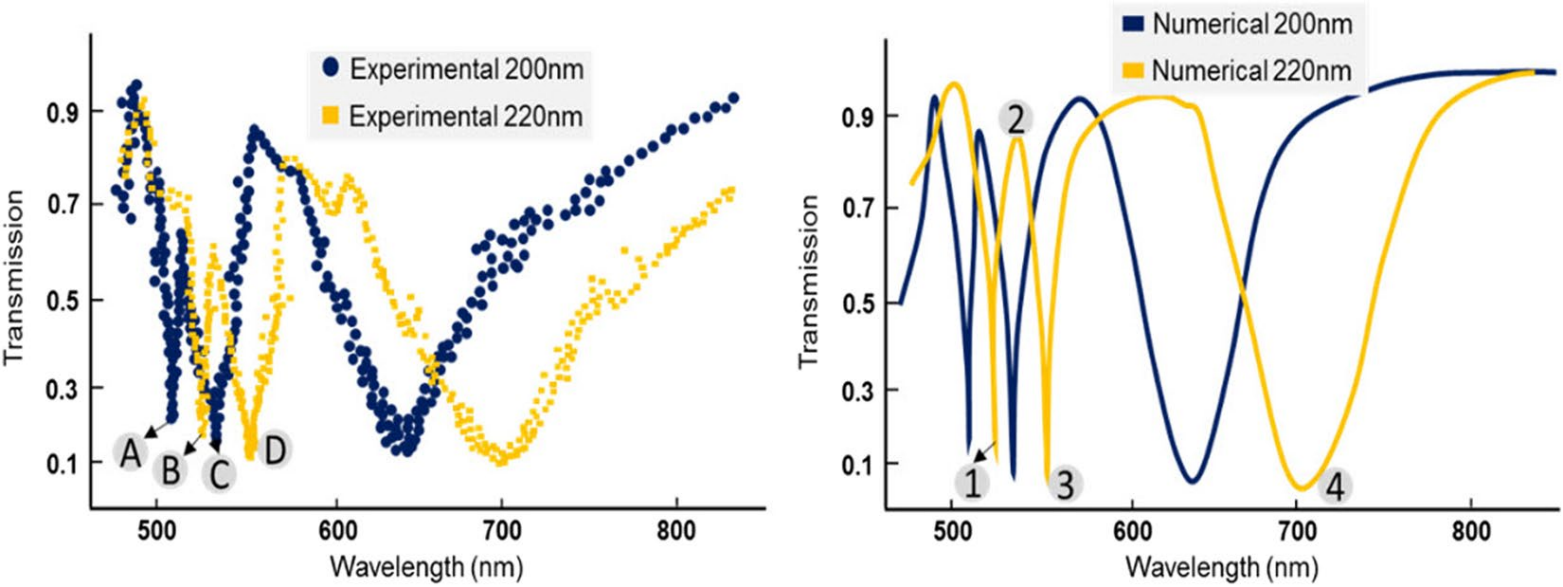
Experimental and numerical transmission spectrum of the proposed substrates with p = 200 nm and p = 220 nm. Resonances occur experimentally at 508 nm, 536 nm, 641 nm, for p = 200 nm, and 529 nm, 555 nm, 707 nm, for p = 220 nm. These values are calculated using the fourth order Lagrange interpolating polynomials.

Atomic force microscopy (AFM) surface topography images revealed an average surface roughness of 1.6 nm as shown in Fig. 5. Figure 6 represents the experimental and full-wave numerical results of the transmission spectrum of the plasmonic substrate for p = 200 nm and p = 220 nm. A TM polarized incident light illuminates the substrate with $\theta =83$°. The transmission spectrum is composed of three resonances located at 508 nm (~590 THz), 536 nm (~556 THz), 641 nm (~468 THz), for p = 200 nm, and 529 nm (~567 THz), 555 nm (~540 THz), 707 nm (~424 THz), for p = 220 nm, respectively. A good agreement between the experimental and numerical results can be observed.

**Figure 7:**
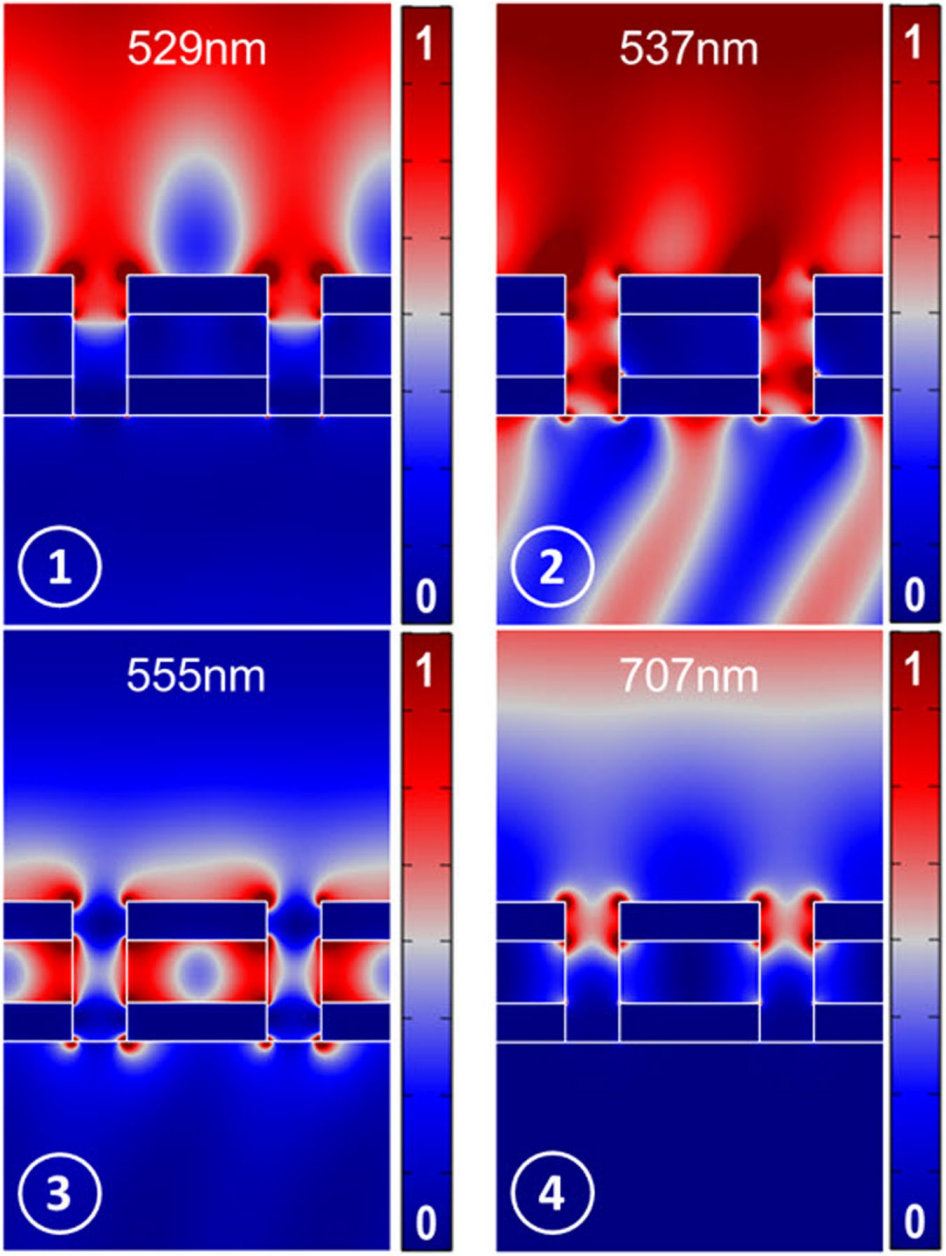
Calculated electric field intensity (|E|^2^/|E_0_|^2^) at the resonance wavelengths labeled in Fig. 5 from 1 through 4, respectively. Strong coupling between surface plasmon polaritons and plasmonic cavity resonances at resonance frequencies can be observed.

The measured optical transmission results indicate ultra-sharp bandgaps at the resonance wavelengths. The corresponding *Q*-factors based on the full-width-at-half-maximum (defined as ${{\lambda }}_{{r}{e}{s}}/{F}{W}{H}{M}$ ^49,50^) extracted from the experimental data for the first four resonances (labeled with A, B, C, and D) are 39.6, 34.7, 26.1, and 24.5, respectively, which are remarkable in plasmonic resonances. For an intuitive notion of high-*Q* bandgap formation, surface plasmon excitations on the substrate with p = 220 nm at the resonance frequencies are presented in Fig. 7. As a result of gap plasmon excitation at 529 nm resonance wavelength, a strong electric field is confined on the surface of the substrate. At 555 nm, surface plasmons at the interface between the silver layers and air are excited. In addition, it can be seen that the plasmons inside the MIM cavity have been excited. Intense electric field is also confined in the optical cavity at 707 nm which instigates near-unity reflection. Extreme coupling between the localized cavity mode plasmons and the surface plasmon polaritons in the structure at the resonance wavelengths can be recognized. Magnetic field intensity and power flow are shown in *Supplementary S4*.

**Figure 8:**
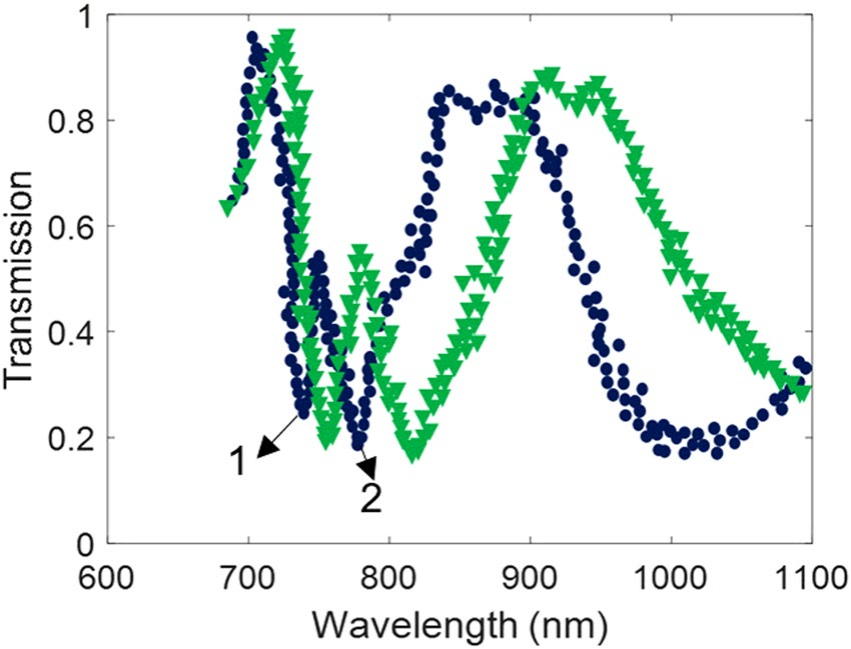
Sensitivity measurement with p = 200 nm substrate. Sensitivity of about 674 nm/RIU for the first, and 1245 nm/RIU for the second resonances were observed.

As pointed out, the proposed structure can be used in a variety of plasmon-enhanced applications including biosensing, optical imaging, and SERS due to its sharp optical transmission response. In order to validate the applicability of the proposed substrate, refractive index sensing of the surrounding medium was performed. To precisely control the index of the surrounding dielectric medium (down to 0.01), we applied commercially available index matching fluids (Cargille Labs). Figure 8 shows sensing results by changing the refractive index of the surrounding medium from 1.33 (water) to 1.38.

## Summary and Conclusion

In summary, we presented plasmonic substrates based on metal-insulator-metal (MIM) stacks with ultra-sharp optical transmission responses for plasmonic-enhanced applications. Through engineering the geometrical parameters, excitation angle, and cavity mode resonances, we were able to achieve remarkably sharp plasmonic resonances. To investigate the formation of high-*Q* bandgaps in thin metallic films at the Visible and near-IR regions, we carried out a parametrical study to inspect the effect of metal/dielectric thickness, insulator permittivity, and the incident angle. The angular sharpness of the plasmonic resonances significantly depend on the angle of incidence. Experimental refractive index sensing results shows bulk wavelength sensitivity as high as 1245 nm per refractive index unit (nm/RIU) with no surface functionalization. Acquired sensitivity results are exceptionally high compared to the most plasmonic based nanostructure and planer metamaterial sensors^51–53^. The presented bandgap mechanism may be applied to the development of novel nanophotonic filters, such as reflection color filters and biomedical sensing devices with added tunability for high-throughput screening.

## Methods

### Sample fabrication

Initially, commercially available BK7 glass substrate was cleaned off via “piranha (H_2_SO_4_ + H_2_O_2_) solution” cleaning procedure to completely remove organic residues on the substrate. The cleaned substrate was then completely dried on the hot plate at 200 °C and then allowed to cool. Secondly, the MIM Ag/SiO_2_/Ag film with 50 nm/80 nm/50 nm was evaporated on the substrate aided by E-beam. Note that 2.5 nm thick Ge layer was used to serve as a wetting layer to enhance adhesion and surface quality of MIM film. An array of nanogratings for different periods (200 nm and 220 nm) were defined in MIM film by focused ion beam (with a gallium ion current of 9.7 pA and an accelerating voltage of 30 KeV) milling. The milled slit had a width of 55–70 nm supporting the fundamental modal propagation inside.

### Optical measurement setup

We characterize the optical transmission spectra of all samples with a custom optical setup allowing the coverage and measurement of the spectrum of a wide-angle incident light. The index matching fluid (n = 1.52) was dispensed to seal the gap between the objective lens and the substrate to maximize the acceptance angle of the objective lens. The transmission spectrum was then resolved by diffraction grating (150 g/mm with blaze wavelength of 500 nm) and recorded on linear array CCD (PIXIS, Princeton Instruments), shown in Fig. 6. For the wide-angle illumination of device, Abbe condenser (1.25 N.A.) coupled with broadband light source (halogen/tungsten lamp) was used.

### Numerical simulations

Finite element method (FEM) simulations are performed to obtain the optical reflection spectra and field distributions using the COMSOL Multiphysics software. Periodic boundary conditions are employed along the *x* axis to account for the periodic arrangement of the unit cells. Measured values of the permittivity of Ag (imaginary part multiplied by factor of 1.5 to account for surface irregularities and additional losses induced by fabrication) and SiO2 were used. The Silver and Quartz optical constants were modelled using a COMSOL fit to data collected by Palik. Analytical transmission line modeling results were implemented by the homemade programs in MATLAB^®^ environment.

## Electronic supplementary material

Supplementary Information
