# Supplementary material for: Tunable plasmonic substrates with ultrahigh Q-factor resonances
Source: Sci Rep. 2017 Nov 22;7:15985. doi: 10.1038/s41598-017-16288-3 (PMC5700073; doi:10.1038/s41598-017-16288-3)
Supplement: Supplementary file 1 — Supplementary Information [file 41598_2017_16288_MOESM1_ESM.pdf]

Supplementary Information

Tunable plasmonic substrates with ultrahigh *Q*-factor resonances

Hamid T. Chorsi[1], Youngkyu Lee[2], Andrea Alù[2] and John X.J. Zhang[1,*]

[1]Thayer School of Engineering, Dartmouth College, Hanover, NH 03755, USA

[2]Electrical and Computer Engineering, University of Texas at Austin, Texas 78712, USA

1. The electric and magnetic field intensity of the double-layer structure in Fig. 2

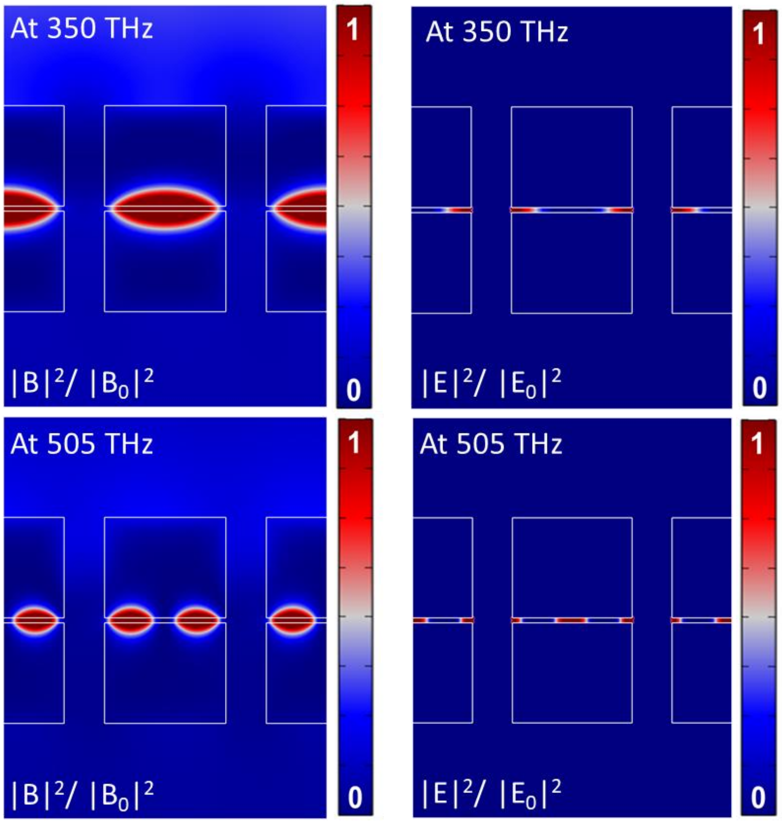

Figure S1. Electric and magnetic field intensity results for a double-layer plasmonic grating in air with $w_S = 40$ nm, $t_M = 100$ nm, $p = 180$ nm, and $w_D = 5$ nm.

Fig. S1 presents the electric (right-hand side column) and magnetic (left-hand side column) intensity plots of the proposed structure in Fig. 2 with ultra-sharp bandgap response ($w_D = 5$ nm). The top row shows the field intensity results for the fundamental mode at 350 THz and the bottom row represents the field intensity results for the higher-order resonance mode with sharp bandgap response at 505 THz. The nanograting substrate is normally excited with $\theta = 63°$.

2. **The effect of insulator permittivity on the transmission response of the plasmonic nanogratings.**

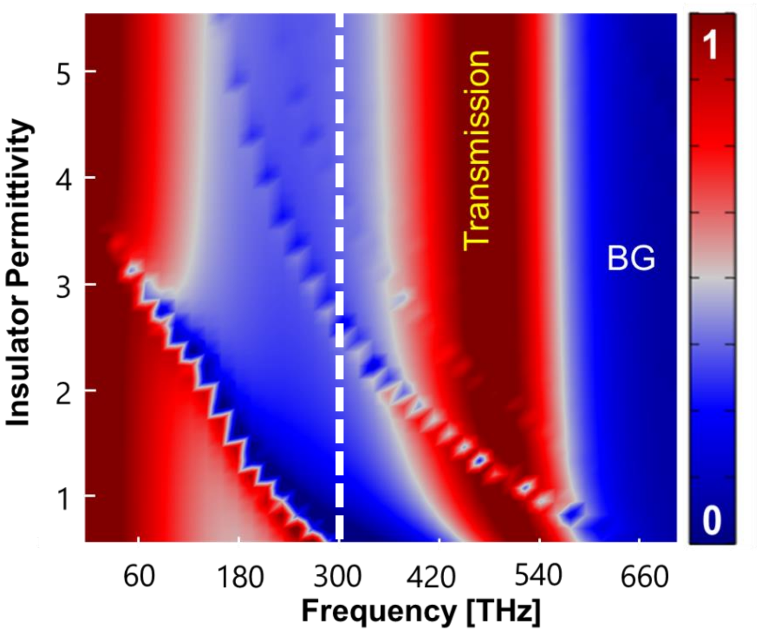

Figure S2. Optical response of the substrate with respect to the insulator permittivity at optical frequency, $\theta = 86°$. The BG response is not considerably affected by increasing or decreasing the insulator permittivity.

The influence of substrate permittivity on the transmission spectrum of the plasmonic substrate with p=180 nm is shown in Fig. S2. It can be seen that the insulator permittivity does not significantly affect the optical transmission response.

3. **Thickness-dependent dispersion of an MIM substrate**

Considering an MIM structure shown in Fig. S3, with a dielectric gap of width D_1 and metal thickness of D_2, the dispersion relation can be obtained through solving the wave equation in Helmholtz form. Please note that the equation considers both the metal and insulator thicknesses, thus can be used for symmetric and asymmetric structures.

$$-\varepsilon_m \frac{D_1}{2}\sqrt{k_{spp}^2-\varepsilon_d k_0^2} =$$

$$\ln\left[\frac{\tanh(\varepsilon_m \frac{D_1^2}{D_2}\sqrt{k_{spp}^2-\varepsilon_m k_0^2})\left[\sqrt{k_{spp}^2-\varepsilon_d k_0^2}\,(k_{spp}^2-\varepsilon_m k_0^2)(1+\mathrm{i})+\sqrt{k_{spp}^2-\varepsilon_b k_0^2}\sqrt{k_{spp}^2-\varepsilon_d k_0^2}\right]+}{\tanh(\varepsilon_m \frac{D_1^2}{D_2}\sqrt{k_{spp}^2-\varepsilon_m k_0^2})\left[\sqrt{k_{spp}^2-\varepsilon_d k_0^2}\,(k_{spp}^2-\varepsilon_m k_0^2)(-1+\mathrm{i})+\sqrt{k_{spp}^2-\varepsilon_b k_0^2}\sqrt{k_{spp}^2-\varepsilon_d k_0^2}\right]+}\right.$$

$$\left.\frac{\sqrt{k_{spp}^2-\varepsilon_d k_0^2}\sqrt{k_{spp}^2-\varepsilon_m k_0^2}\,(1+\sqrt{k_{spp}^2-\varepsilon_b k_0^2}+\sqrt{k_{spp}^2-\varepsilon_d k_0^2})+\sqrt{k_{spp}^2-\varepsilon_m k_0^2}\,(k_{spp}^2-\varepsilon_m k_0^2)}{\sqrt{k_{spp}^2-\varepsilon_d k_0^2}\sqrt{k_{spp}^2-\varepsilon_m k_0^2}\,(-1-\sqrt{k_{spp}^2-\varepsilon_b k_0^2}+\sqrt{k_{spp}^2-\varepsilon_d k_0^2})+\sqrt{k_{spp}^2-\varepsilon_m k_0^2}\,(k_{spp}^2-\varepsilon_m k_0^2)}\right]$$

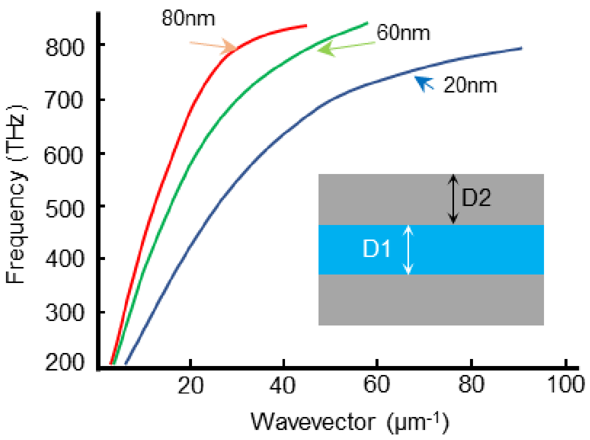

Figure S3. Dispersion relation for an MIM layered medium with the structure in the inset. Three cases include D_1=80 nm, 60 nm, and 20 nm.

The obtained results are in agreement with the numerical and experimental results in [1, 2].

4. Magnetic field intensity and power flow

Calculated corresponding magnetic field intensity and power flow of the fabricated plasmonic substrate with p=220 nm obtained via full-wave solver [3].

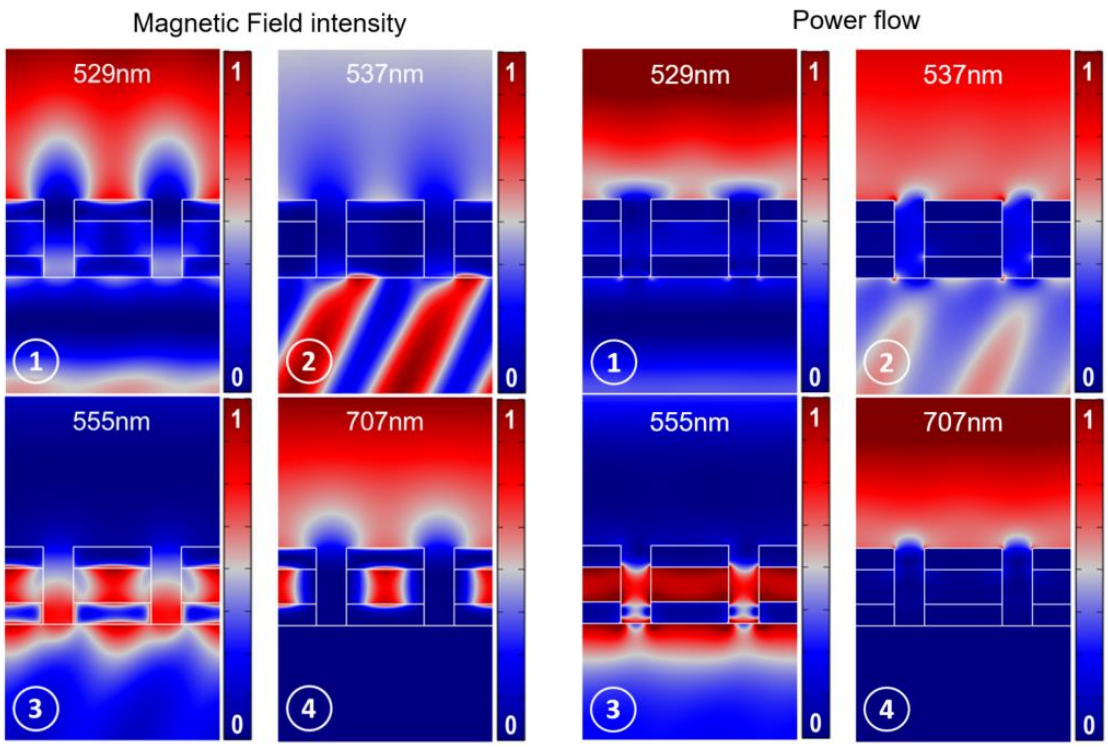

Figure S4. Corresponding magnetic field intensity and power flow for Fig. 7.

References

1. Bozhevolnyi, S.I., October. Plasmonic nano-guides and circuits. Pan Stanford Publishing, (2009).

2. Kuttge, M., Cai, W., de Abajo, F.J.G. and Polman, A., Dispersion of metal-insulator-metal plasmon polaritons probed by cathodoluminescence imaging spectroscopy. Physical Review B, 80(3), p.033409, (2009).

3. Chorsi, Hamid T., Youngkyu Lee, Andrea Alu, and John X. Zhang. "Plasmonic-enhanced Metallic Nanogratings for Ultrahigh Q-factor Resonances." Novel Optical Materials and Applications. Optical Society of America, 2017.
